# Aminothiazoles inhibit osteoclastogenesis and PGE_2_ production in LPS‐stimulated co‐cultures of periodontal ligament and RAW 264.7 cells, and RANKL‐mediated osteoclastogenesis and bone resorption in PBMCs

**DOI:** 10.1111/jcmm.14015

**Published:** 2018-12-01

**Authors:** Anna Kats, Natalija Gerasimcik, Tuomas Näreoja, Jonas Nederberg, Simon Grenlöv, Ekaterina Lagnöhed, Suchita Desai, Göran Andersson, Tülay Yucel‐Lindberg

**Affiliations:** ^1^ Department of Dental Medicine, Division of Periodontology Karolinska Institutet Huddinge Sweden; ^2^ Department of Laboratory Medicine, Division of Pathology Karolinska Institutet, Karolinska University Hospital Huddinge Sweden

**Keywords:** aminothiazoles, bone resorption, human PBMCs, lipopolysaccharide, microsomal prostaglandin E synthase‐1, osteoclastogenesis, osteoclasts, periodontal ligament cells, prostaglandin E_2_, RAW 264.7 cells

## Abstract

Inflammatory mediator prostaglandin E_2_ (PGE_2_) contributes to bone resorption in several inflammatory conditions including periodontitis. The terminal enzyme, microsomal prostaglandin E synthase‐1 (mPGES‐1) regulating PGE_2_ synthesis is a promising therapeutic target to reduce inflammatory bone loss. The aim of this study was to investigate effects of mPGES‐1 inhibitors, aminothiazoles TH‐848 and TH‐644, on PGE_2_ production and osteoclastogenesis in co‐cultures of periodontal ligament (PDL) and osteoclast progenitor cells RAW 264.7, stimulated by lipopolysaccharide (LPS), and bone resorption in RANKL‐mediated peripheral blood mononuclear cells (PBMCs). PDL and RAW 264.7 cells were cultured separately or co‐cultured and treated with LPS alone or in combination with aminothiazoles. Multinucleated cells stained positively for tartrate‐resistant acid phosphatase (TRAP) were scored as osteoclast‐like cells. Levels of PGE_2_, osteoprotegerin (OPG) and interleukin‐6, as well as mRNA expression of *mPGES‐1, OPG* and *RANKL* were analysed in PDL cells. PBMCs were treated with RANKL alone or in combination with aminothiazoles. TRAP‐positive multinucleated cells were analysed and bone resorption was measured by the CTX‐I assay. Aminothiazoles reduced LPS‐stimulated osteoclast‐like cell formation both in co‐cultures and in RAW 264.7 cells. Additionally, aminothiazoles inhibited PGE_2_ production in LPS‐stimulated cultures, but did not affect LPS‐induced *mPGES‐1, OPG* or *RANKL* mRNA expression in PDL cells. In PBMCs, inhibitors decreased both osteoclast differentiation and bone resorption. In conclusion, aminothiazoles reduced the formation of osteoclast‐like cells and decreased the production of PGE_2_ in co‐cultures as well as single‐cell cultures. Furthermore, these compounds inhibited RANKL‐induced bone resorption and differentiation of PBMCs, suggesting these inhibitors for future treatment of inflammatory bone loss such as periodontitis.

## 1 INTRODUCTION

In chronic inflammatory conditions including periodontitis and rheumatoid arthritis, bone loss is initiated by unresolved inflammation in the neighbouring tissues leading to pathological osteoclastogenesis.^1^ During osteoclastogenesis, osteoclast progenitors, recruited from the monocytic linage, differentiate into pre‐osteoclasts, fuse and form large multinucleated cells and finally mature into activated osteoclasts that start to express tartrate‐resistant acid phosphatase (TRAP) and release acid and collagen‐degrading enzymes resulting in bone resorption.^1^, ^2^

Osteoclastogenesis is regulated by a broad range of molecules and stimuli, including cytokines, prostaglandins and growth factors, which directly/indirectly regulate the production of osteoclastogenic molecules by various cell types, including stromal cells, osteoblast, osteocytes or resident fibroblasts.^1^ The main signalling molecules regulating osteoclast formation are macrophage colony stimulating factor (M‐CSF), receptor activator of nuclear factor κB (RANK), RANK ligand (RANKL) and osteoprotegerin (OPG). RANKL and OPG are mainly produced by osteoblasts, osteocytes, resident cells such as periodontal ligament (PDL) cells, and in case of inflammation also by activated T‐cells.^1^ Whereas OPG is the main osteoclastogenesis‐inhibitory molecule, specifically increasing bone density, RANK‐RANKL and M‐CSF are main molecules inducing bone resorption.^1^, ^3^ The latter can also be induced through periodontal bacteria or bacterial components such as lipopolysaccharide (LPS) acting via Toll‐like receptors (TLRs) 4 and 2, leading to increased expression of RANKL and other proinflammatory cytokines and osteoclastogenesis‐stimulating molecules, including TNFα, interleukin (IL)‐1 and M‐CSF.^1^, ^3^ In vitro experiments have reported that PDL cells, mainly producing OPG, adapt to bacterial stimuli by producing increased levels of osteoclastogenesis‐stimulating molecules, thus increasing bone resorption.^3^

The inflammatory mediator prostaglandin E_2_ (PGE_2_) may affect inflammation‐induced osteoclastogenesis by stimulation of osteoblasts/stromal cells or by direct stimulation of osteoclast progenitor cells.^4^ PGE_2_ is synthesised sequentially by the enzymes phospholipase A_2_, cyclooxygenases (COX‐1 and COX‐2) and the final prostaglandin E synthases (PGES).^4^ Three isoforms of PGES have been identified; the inducible microsomal PGES‐1 (mPGES‐1), and the constitutively expressed membrane‐associated PGES‐2 and cytosolic PGES.^5^, ^6^, ^7^, ^8^, ^9^

In the chronic inflammatory disease periodontitis, elevated levels of PGE_2_ have been reported in gingival crevicular fluid of patients with periodontitis suggesting this mediator as a predictor of alveolar bone loss.^10^, ^11^ Increased levels of PGE_2_ correlate well with enhanced levels of COX‐2 and mPGES‐1, expressed also in inflamed gingival tissues.^9^, ^11^ Controlling the production of PGE_2_ through COX‐inhibition, using non‐steroidal anti‐inflammatory drugs (NSAIDs) or selective COX‐2 inhibitors, have been reported to decrease the progression of alveolar bone loss and gingival inflammation in periodontitis.^11^, ^12^, ^13^, ^14^ However, long‐term treatment with NSAIDs and selective COX‐2 inhibitors is associated with adverse side effects such as gastrointestinal toxicity and increased the risk of drug‐induced heart failure.^15^, ^16^, ^17^, ^18^, ^19^, ^20^ The downstream enzyme mPGES‐1, responsible for PGE_2_ production, is therefore an attractive target for new anti‐inflammatory drugs with potentially fewer side effects compared to COX‐inhibitors.^21^ However, to the best of our knowledge, mPGES‐1 inhibitors are not yet available for clinical use.

The aminothiazoles TH‐848 and TH‐644 have been identified as mPGES‐1 inhibitors by docking models towards the three‐dimensional crystal structure of mPGES‐1.^22^ These compounds inhibit cytokine‐induced PGE_2_ synthesis in gingival fibroblasts and in vitro enzyme activity of mPGES‐1 as well as reduce alveolar bone resorption in vivo.^22^ In addition, the aminothiazoles inhibited both RANKL‐ and LPS‐stimulated osteoclast formation of the osteoclast progenitor cell line RAW 264.7.^23^ In the present study, we aimed to further elucidate the effect of aminothiazoles on (a) osteoclastogenesis and the production of PGE_2_, IL‐6, OPG and mPGES‐1 in a co‐culture model using PDL and RAW 264.7 cells stimulated by LPS and (b) osteoclastogenesis and bone resorption in peripheral blood mononuclear cells (PBMCs) stimulated with RANKL.

## 2 MATERIALS AND METHODS

### 2.1 PDL cells

Periodontal ligament cells were obtained from the root surface of extracted teeth without any clinical signs of periodontal disease from three healthy donors and isolated as previously described.^24^ The study was approved by the Regional Ethical Review Board Stockholm and informed consent was obtained from the donors. The cells were harvested from the primary outgrowth by trypsinisation and cultured in Dulbecco's minimal essential medium supplemented with 10% foetal bovine serum (FBS, heat‐inactivated), penicillin (50 U/mL) and streptomycin (50 μg/mL) (all from Invitrogen Life Technologies, Waltham, MA, USA) in 37°C at 100% humidity in 5% CO_2_. For the experiments, PDL cells were either seeded in 24‐well plates (20 000 cells/well) in a volume of 0.5 mL or in cell culture dishes (500 000 cells/dish) in a volume of 1.8 mL and treated with *Escherichia coli* LPS (1 μg/mL; Sigma‐Aldrich, St. Louis, MO, USA) alone or in combination with aminothiazoles 4‐([4‐(2‐naphthyl)‐1,3‐thiazol‐2‐yl]amino)phenol (TH‐848; 0.2 μmol/L) or 4‐(3‐fluoro‐4‐methoxyphenyl)‐*N*‐(4‐phenoxyphenyl)‐1,3‐thiazol‐2‐amine hydrobromide (TH‐644; 2 μmol/L) (Chem‐Bridge Corp., San Diego, CA, USA). The cells were collected for isolation of mRNA after 24 hours of treatment. For PGE_2_, OPG and IL‐6 analysis, the medium from the 24‐well plates was collected after 48 hours and stored in −20°C.

### 2.2 RAW 264.7 cells

RAW 264.7 cells (ATCC, Manassas, VA, USA) were cultured in α‐minimal essential medium (α‐MEM; Sigma‐Aldrich) supplemented with 10% FBS, l‐glutamine (2 mmol/L), penicillin (50 U/mL) and streptomycin (50 μg/mL) (all from Invitrogen Life Technologies) in 37°C at 100% humidity in 5% CO_2_ as previously described.^25^

For the experiments, RAW 264.7 cells were seeded in 24‐well plates (5000 cells/well) and treated with LPS (1 μg/mL), alone or in combination with aminothiazoles TH‐848 (0.2 μmol/L) or TH‐644 (15 μmol/L) in a total volume of 0.5 mL. After 2 days, the medium containing all the reagents described above was replaced and stored in −20°C for later PGE_2_, OPG and IL‐6 analysis. The cells were allowed to further differentiate into osteoclast‐like cells; and after 4 days, the experiment was stopped, the cells were fixed in 2% formaldehyde and stained for TRAP (Supporting Information).

### 2.3 Co‐culture experiments

Periodontal ligament cells (5000 cells/well) and RAW 264.7 (5000 cells/well) were seeded in α‐MEM in 24‐well plates (Nunclon Delta; Thermo Fischer Scientific Nunc A/S, Roskilde, Denmark). Cells were seeded in either direct cell‐cell contact or separated by inserts with a semipermeable membrane (pore size 0.4 μm) enabling the cells to exchange soluble factors. Cells were treated with LPS (1 μg/mL) alone or in combination with the aminothiazoles TH‐848 (0.2 μmol/L) or TH‐644 (15 μmol/L) in a total volume of 1 mL/well. The optimal concentrations of TH‐848 and TH‐644 used in the experiments were previously determined by dose‐response experiments.^23^ The cells were incubated for 48 hours at 37°C in 100% humidity with 5% CO_2_ and 800 μL of medium was collected and stored in −20°C for PGE_2_, OPG and IL‐6 analysis. Thereafter, the cells were re‐stimulated with 800 μL medium containing the same reagents as described above to further allow osteoclast differentiation of RAW 264.7 cells. After 4 days in total, the experiment was stopped and the cell layers were fixed in 2% formaldehyde and stained for the osteoclast marker TRAP (Supporting Information).

### 2.4 Differentiation of PBMCs

Peripheral blood mononuclear cells were purified from buffy coats donated by five healthy males who had given their informed consent (Karolinska University Hospital Laboratory, Huddinge, Sweden) using density gradient centrifugation on Ficoll Paque Plus (GE Healthcare, Uppsala, Sweden). For more uniform differentiation of osteoclasts,^26^ CD14‐positive fraction of PBMCs was selected with CD14^+^ magnetic microbeads, according to the manufacturer's instructions (Miltenyi Biotec, Bergisch Gladbach, Germany). The CD14^+^ PBMCs were cultured in 96‐well plates (NUNC, delta surface) in α‐MEM, supplemented with 10% FBS, GlutaMAX (2 mmol/L), streptomycin (100 μg/mL), penicillin (100 U/mL) (all from GIBCO, Grand Island, NY, USA), either on plastic surface (150 000 cells/well) or on 130 μm thick bovine cortical bone slices (250 000 cells/well) and differentiated into multinuclear cells for 6‐12 days in the presence of recombinant human M‐CSF (30 ng/mL; R&D Systems, Minneapolis, MN, USA) and sRANKL (2 ng/mL; Peprotech, Rocky Hill, NJ, USA) with or without aminothiazoles TH‐848 (0.2 μmol/L) or TH‐644 (2 or 15 μmol/L) (Chem‐Bridge Corp.). Half of the medium was changed every 3 days and samples were collected from the plastic wells on day 6 and from the bone slices on days 9 and 12. To minimise proteolysis, cysteine protease inhibitor E64 (10 ng/mL; Sigma‐Aldrich) was added to the medium samples, which were used for analyses of bone resorption. The cells from the plastic wells and bone slices were stained for TRAP, as described in Supporting Information.

### 2.5 Bone resorption

The TRAP‐stained bone slices from day 12 were imaged in coverslip‐bottomed chambers, immersed in H_2_O, using 60x water immersion objective and NikonA1+ confocal laser microscope system (Nikon, Tokyo, Japan). We used reflection signal obtained with 405 nm laser to image bone contour and 647 nm laser to excite the TRAP stain. To visualise the resorption pits, Z‐stacks of bone contour and TRAP stain were captured and processed to volumetric 3D projections with ImageJ plugin collection Fiji (2.0.0‐rc‐65/1.52b) and to videos scrolling through the stacks (Videos S1‐S4).

To quantify bone resorption, culture medium from the days 9 and 12 was analysed for C‐terminal cross‐linking telopeptides of type I collagen (CTX‐I) using Crosslaps for Culture CTX‐I ELISA kit (AC‐07F1, Ids, Frankfurt am Main, Germany) according to the manufacturer's instructions.

### 2.6 PGE_2_ analysis

The levels of PGE_2_ in the supernatants collected from the cell cultures were analysed using a commercial PGE_2_ enzyme immunoassay (EIA) kit (Cayman Chemicals, Ann Arbor, MI, USA) according to the manufacturer's instructions.

### 2.7 Analysis of OPG and IL‐6

Levels of OPG and IL‐6 were analysed in the culture supernatants collected from co‐cultures as well as cultures of PDL cells alone after 48 hours of treatment. The analysis was performed with Luminex technology on a Bioplex Suspension Array System (Bio‐Rad Laboratories, Hercules, CA, USA) with a commercial human bone Milliplex MAP Kit (EMD Millipore Corporation, Burlington, MA, USA) according to the manufacturer's instructions. The minimum detection levels of the assay were 1.9 pg/mL for OPG and 1.4 pg/mL for IL‐6.

### 2.8 Quantitative RT‐qPCR

Periodontal ligament cells were seeded in Petri dishes (500 000 cells/dish) and cultured as described above. After 24 hours of treatment with LPS in the absence or presence of TH‐848 (0.2 μmol/L) and TH‐644 (2 μmol/L), total RNA was isolated from the cells using the commercial RNeasy Mini Kit (Qiagen, Valencia, CA, USA). The amount of total RNA was quantified using a NanoVue Plus Spectrophotometer (GE Healthcare), and first‐strand cDNA was obtained by reverse transcription of 1 μg of total RNA using the iScript™ cDNA Synthesis Kit (BioRad, Hercules, CA, USA) in a volume of 20 μL. The quantitative polymerase chain reaction (RT‐qPCR) was performed by amplification of 50 ng of cDNA. The gene expression analysis of *mPGES‐1* (*Mm00452105_m1*), *OPG* (*Hs00900358_m1*) and *RANKL* (*Hs00243522_m1*), the PDL cell marker *S100A4* (*Hs00243202_m1*)^27^ and the housekeeping gene glyceraldehyde 3‐phosphate dehydrogenase (GAPDH; *Hs02758991_m1*) were performed by TaqMan Gene Expression Assays together with Taq‐Man Universal PCR Master Mix (all from Applied Biosystems, Foster City, CA, USA) in a volume of 20 μL. All RT‐qPCR reactions were run in triplicates on the 7500 Fast Real‐Time PCR system (Applied Biosystems). Gene expression was calculated according to the ΔΔCt method, where each sample was normalised to the mean of GAPDH (reference gene).

### 2.9 Statistical analysis

One‐ or two‐way anova with Tukey's or Sidak's corrections for multiple analyses were used to determinate statistical significance between treatment groups. Differences in treatment outcome were considered significant if *P *≤* *0.05.

## 3 RESULTS

### 3.1 Aminothiazoles decrease the formation of osteoclast‐like cells in co‐cultures of PDL and RAW 264.7 cells

**Figure 1:**
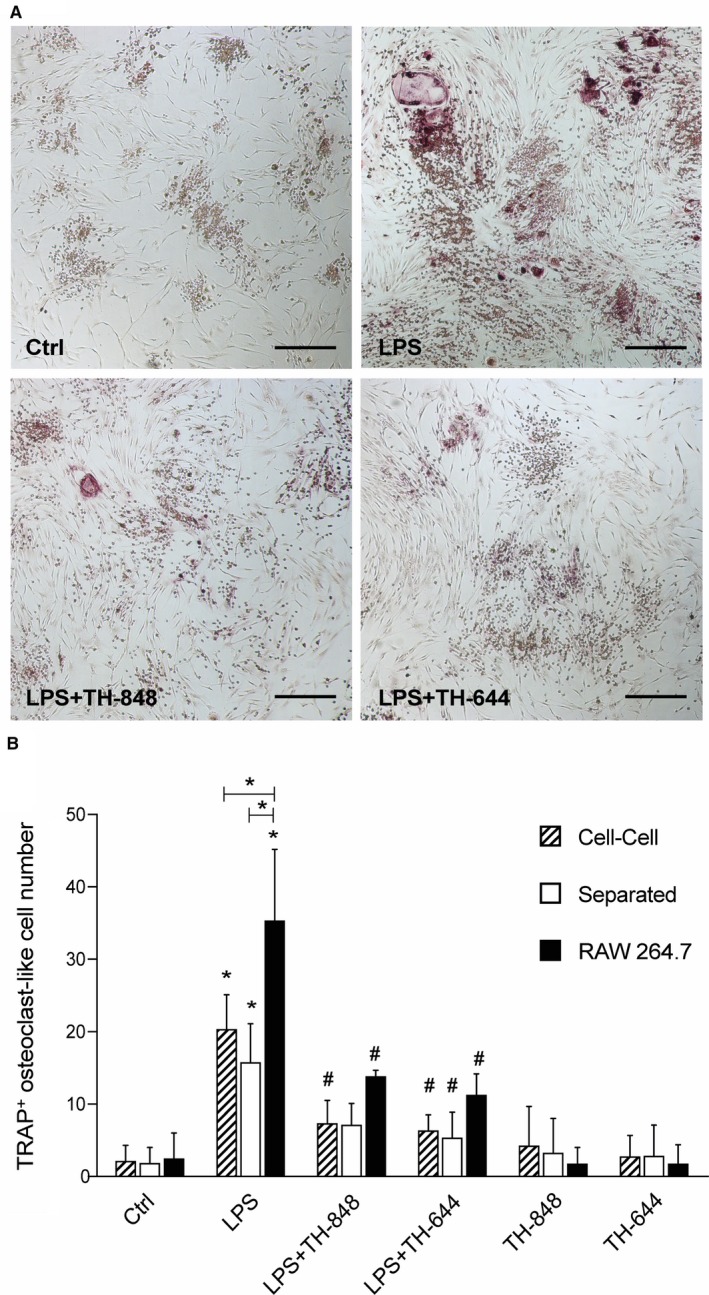
Osteoclast‐like cell formation in co‐cultures of periodontal ligament (PDL) and RAW 264.7 cells. (A) Cell‐cell co‐cultures of PDL and RAW cells stained for tartrate‐resistant acid phosphatase (TRAP). The cells were treated for 4 days with medium (Ctrl) or lipopolysaccharide (LPS; 1 μg/mL), in the absence or presence of aminothiazoles TH‐848 (0.2 μmol/L) or TH‐644 (15 μmol/L). Results shown are representative from three independent experiments. Scale bars = 100 μmol/L. (B) Number of TRAP‐positive osteoclast‐like cells in co‐cultures (cell‐cell or separated by semipermeable membrane) of PDL and RAW cells as well as RAW cells alone treated for 24 h with medium (Ctrl) or LPS, in the absence or presence of aminothiazoles TH‐848 or TH‐644. Data shown are representative of three independent experiments analysed in duplicates or triplicates. Osteoclast‐like cells were counted by two independent observers. *P ≤* *0.05 (compared to Ctrl or between the groups within the treatment); ^#^ *P *≤* *0.05 (compared to LPS)

In agreement with previous findings, LPS treatment stimulated the formation of TRAP‐positive multinucleated cells compared to control cells in cultures of RAW 264.7 cells alone.^23^ Similarly, but to a lower extent, the number of TRAP‐positive osteoclast‐like cells was increased by LPS in cell‐cell co‐cultures and separated co‐cultures of PDL and RAW 264.7 cells (Figure 1A). The number of osteoclast‐like cells, induced by LPS was higher in the RAW 264.7 cells cultured alone compared to co‐cultures with cell‐cell contact (*P *=* *0.075) as well as to co‐cultures separated by semipermeable membranes (*P *=* *0.038) (Figure 1B). When comparing the two co‐culture models, the number of osteoclast‐like cells, induced by LPS, did not significantly differ (Figure 1B). Addition of the aminothiazoles, both TH‐848 (0.2 μmol/L) and TH‐644 (15 μmol/L), decreased osteoclast formation in co‐cultures of PDL and RAW 264.7 cells in cell‐cell contact and in separated co‐cultures (Figure 1B). Furthermore, the addition of the aminothiazoles in combination with LPS decreased the number of osteoclast‐like cells (approximately 2‐to‐3‐fold) in all LPS‐stimulated cultures compared to the cultures treated with LPS alone (Figure 1B).

To verify the origin of PDL cells, we analysed the expression of *S100A4*, which have previously been suggested as a specific marker for these cells.^27^ All PDL cells used in this study were positive for *S100A4* (Figure S1).

### 3.2 Aminothiazoles inhibit PGE_2_ in co‐cultures of PDL and RAW 264.7 cells

**Figure 2:**
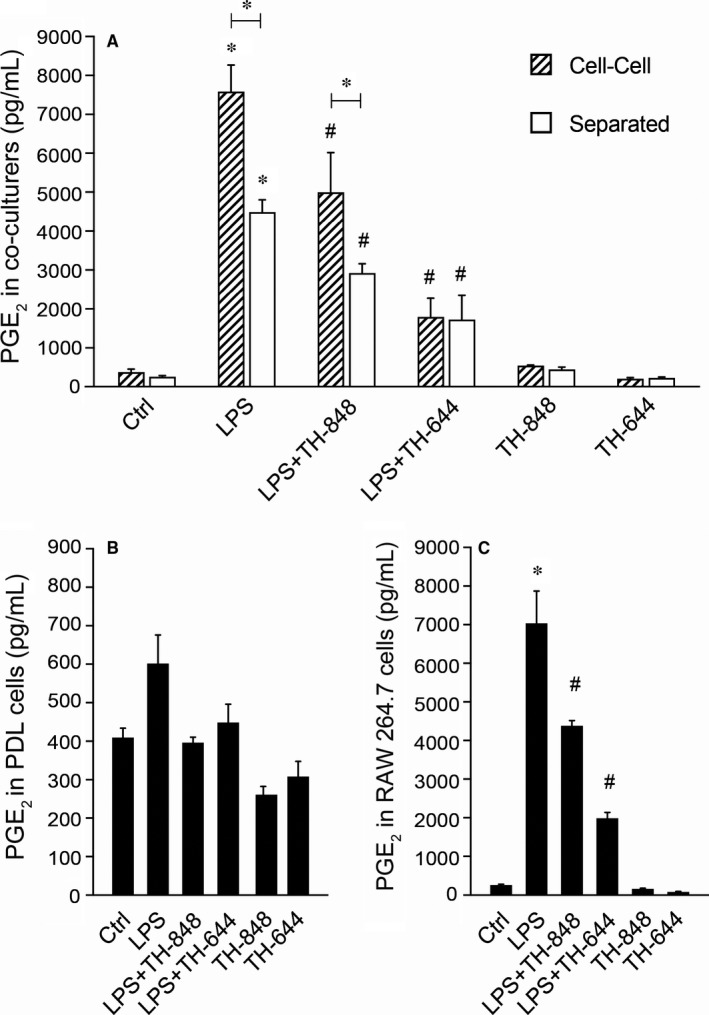
Prostaglandin E_2_ (PGE_2_) production (pg/10 000 cells) in cell‐cell co‐cultures as well as co‐cultures separated by a semipermeable membrane of RAW 264.7 and periodontal ligament (PDL) cells, PDL or RAW 264.7 cells alone (A, B and C, respectively). (A‐C) The cells were treated for 24 h with medium (Ctrl) or lipopolysaccharide (LPS; 1 μg/mL), in the absence or presence of aminothiazoles TH‐848 or TH‐644 in cell‐cell cultures of RAW and PDL cells or separated co‐cultures (A), in single cultures of PDL (B) or RAW cells (C). The inhibitor TH‐848 was used in concentration of 0.2 μmol/L in all cell cultures and for TH‐644, 15 μmol/L was used for co‐cultures and RAW cells and 2 μmol/L for PDL cells. Results shown are representative from three independent experiments, analysed in duplicates or triplicates. *P ≤* *0.05 (compared to Ctrl or between the groups within the treatment); ^*#*^ *P *≤* *0.05 (compared to LPS)

Lipopolysaccharide stimulated the production of PGE_2_ in cell‐cell and separated co‐cultures (Figure 2A) as well as in cultures of PDL (Figure 2B) and RAW 264.7 cells alone (Figure 2C). In LPS‐stimulated co‐cultures of PDL and RAW 264.7 cells, the PGE_2_ levels were significantly (*P *=* *0.002) higher in direct cell‐cell cultures compared to separated co‐cultures (Figure 2A). Addition of aminothiazoles decreased significantly the stimulatory effect of LPS on PGE_2_ production in both co‐cultures (Figure 2A) and in cultures of PDL and RAW 264.7 cells alone (Figure 2B,C). When comparing the levels of PGE_2_ in different cultures, PDL cells alone produced approximately 10‐fold lower PGE_2_ than co‐cultures as well as RAW 264.7 cultures alone (Figure 2).

### 3.3 Aminothiazoles do not affect IL‐6 or OPG production in co‐cultures of PDL and RAW 264.7 cells

**Figure 3:**
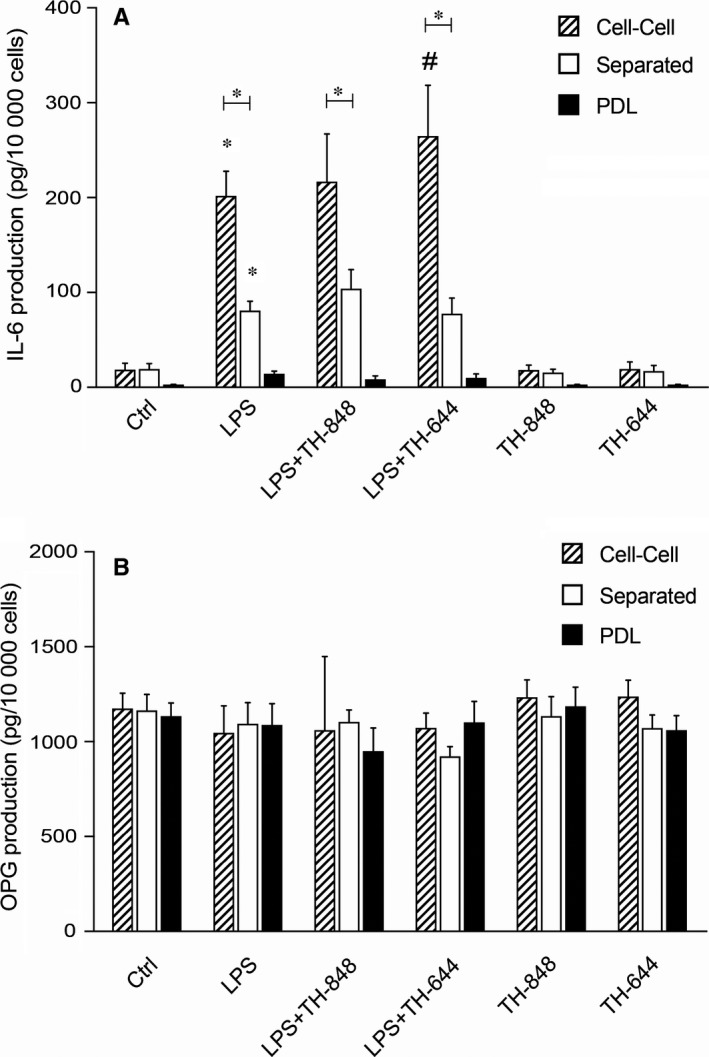
Interleukin (IL)‐6 and osteoprotegerin (OPG) production (pg/10 000 cells) in cell‐cell co‐cultures, co‐cultures of RAW 264.7 and periodontal ligament (PDL) cells separated by a semipermeable membrane, and PDL cells alone (A and B, respectively). The cells were cultured for 24 h with medium (Ctrl) or lipopolysaccharide (LPS; 1 μg/mL), in the absence or presence of aminothiazoles TH‐848 or TH‐644. The inhibitor TH‐848 was used in concentration of 0.2 μmol/L in all cell cultures and for TH‐644, 15 μmol/L was used for co‐cultures and 2 μmol/L for PDL cells. Results shown are representative from three independent experiments, analysed in duplicates or triplicates. *P ≤* *0.05 (compared to Ctrl or between the groups within the treatment); ^*#*^ *P *≤* *0.05 (compared to LPS)

The levels of the inflammatory cytokine IL‐6 were analysed in the supernatants from the PDL and RAW 264.7 co‐cultures as well as from PDL cells alone. LPS stimulated the production of IL‐6 in all cultures, although the concentrations of IL‐6 were 2.5 times higher in cell‐cell compared to separated co‐cultures and 14 times higher as compared to PDL cells alone (Figure 3A). The addition of aminothiazoles in combination with LPS did not significantly affect the levels of IL‐6 induced by LPS (Figure 3A).

The levels of OPG, the RANKL decoy receptor, were also analysed in the same culture supernatants. The results showed that the production of OPG was not affected either by LPS or the aminothiazoles in the cultures (Figure 3B).

### 3.4 Aminothiazoles do not affect the mRNA expression of *mPGES‐1, OPG* or *RANKL* in PDL cells

**Figure 4:**
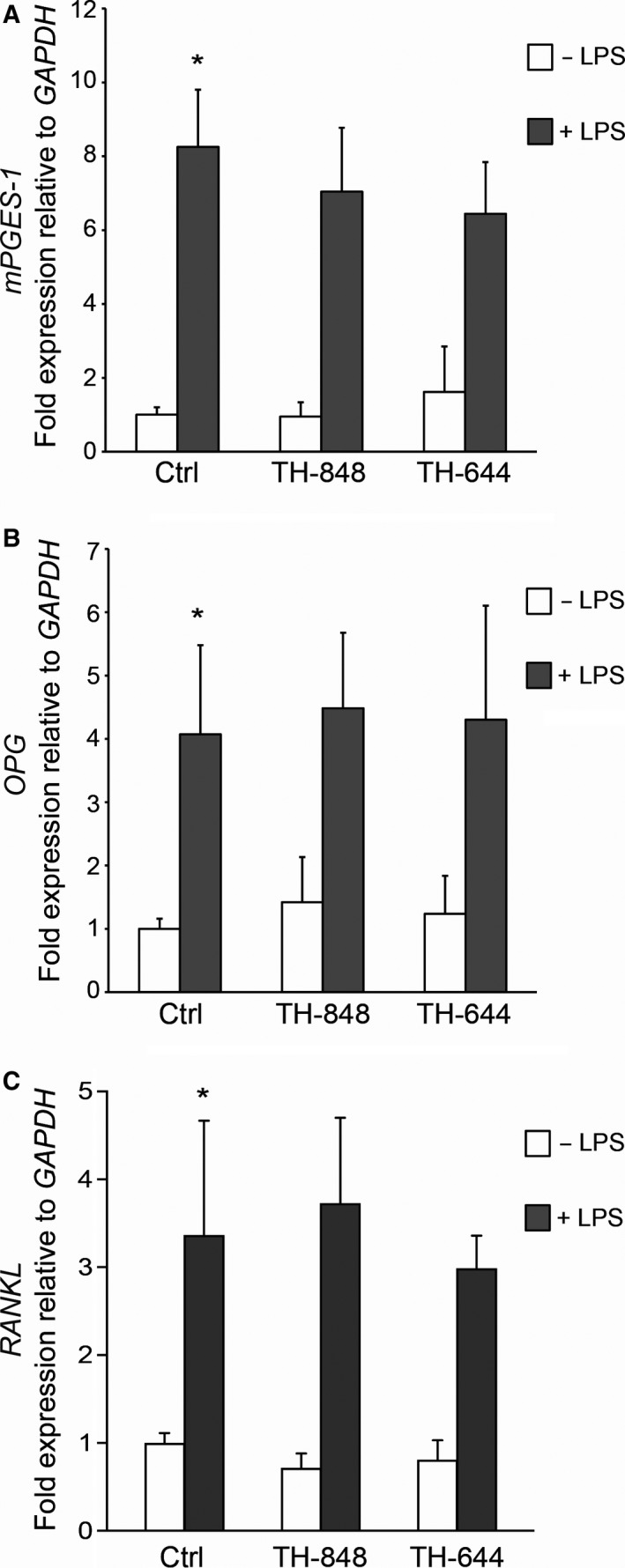
mRNA expression of prostaglandin E synthase‐1 (*mPGES‐1*), osteoprotegerin (*OPG*) and receptor activator of nuclear factor‐κB ligand (*RANKL*) in periodontal ligament (PDL) cells (A, B and C, respectively). Cultures were treated for 24 h with medium (Ctrl) or lipopolysaccharide (LPS; 1 μg/mL), in the absence or presence of aminothiazoles TH‐848 (0.2 μmol/L) or TH‐644 (2 μmol/L). The results are shown as relative mRNA expression and normalised to *GAPDH*±SD, and represents the mean of three independent experiments, analysed in duplicates or triplicates, *P ≤* *0.05 (compared to Ctrl without LPS)

The mRNA expression of *mPGES‐1*,* OPG* and *RANKL* in PDL cells, stimulated by LPS alone or in combination with the aminothiazoles TH‐848 (0.2 μmol/L) or TH‐644 (2 μmol/L), was analysed by RT‐qPCR. The results revealed that *mPGES‐1* mRNA expression was up‐regulated by LPS (Figure 4A). The aminothiazoles, on the other hand, did not affect the LPS‐stimulated *mPGES‐1* expression in PDL cells (Figure 4A). Similar to *mPGES‐1*, the mRNA expression of *OPG* and *RANKL* was up‐regulated by LPS, but not affected by the aminothiazoles (Figure 4B,C, respectively).

### 3.5 Aminothiazoles decrease osteoclastogenesis and bone resorption in PBMC cultures

**Figure 5:**
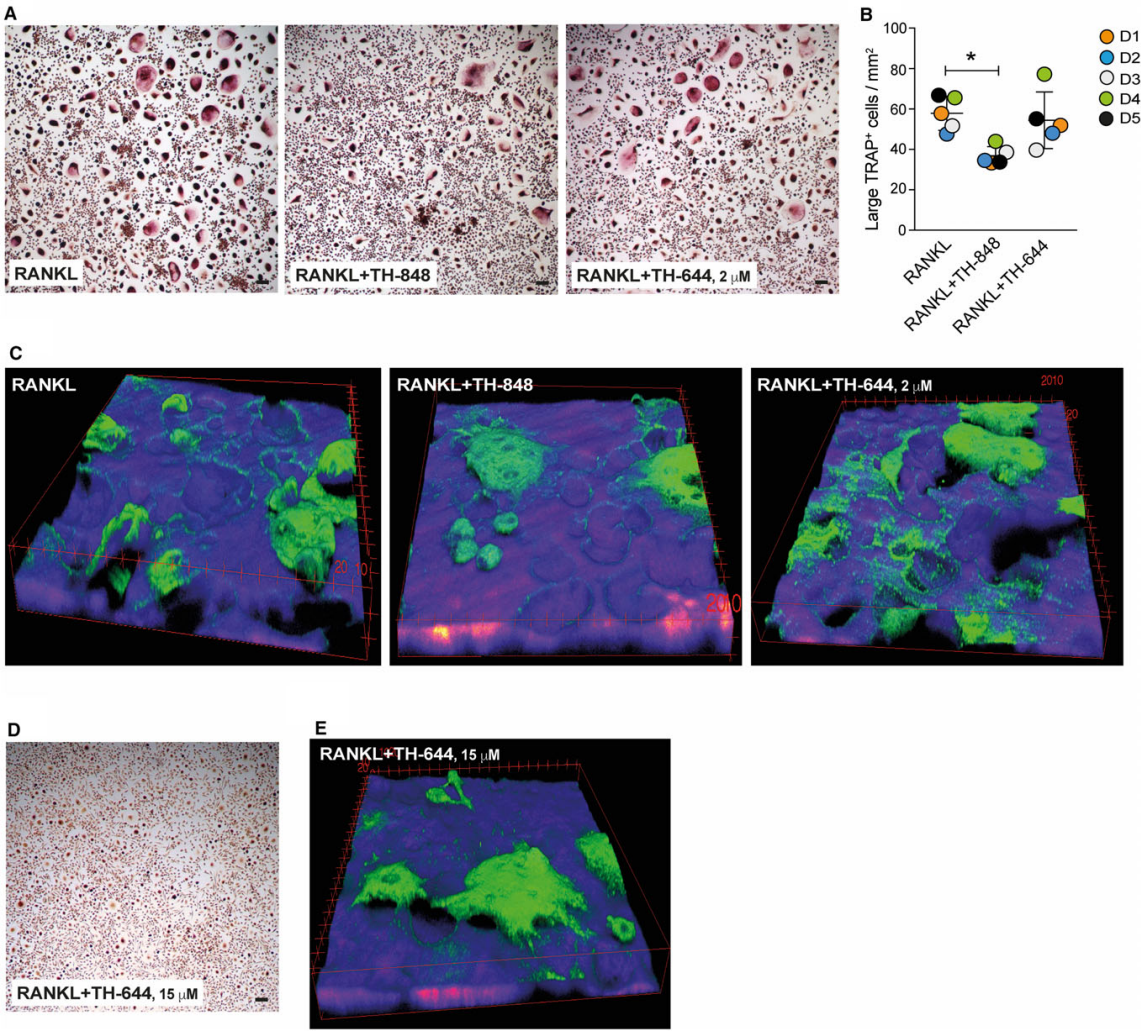
RANKL‐induced osteoclast differentiation from peripheral blood mononuclear cells (PBMCs). The CD14^+^ PBMCs were differentiated with M‐CSF (30 ng/mL) and RANKL (2 ng/mL) alone or in the presence of aminothiazoles TH‐848 (0.2 μmol/L) or TH‐644 (A‐C) (2 μmol/L, n = 5) and (D, E) (15 μmol/L, n = 2) and stained for TRAP on day 6 or 12. (A and D) Representative images of TRAP‐positive osteoclasts on day 6 are shown. Scale bars = 100 μmol/L. (B) Quantification of TRAP‐positive osteoclasts in cultures, showing mean value of quadruplicates, where dots represents the donors (D1‐D5) with mean ± SD, *P ≤* *0.05. (C and E) Representative images of bone resorption pits formed by osteoclasts, differentiated for 12 days on the bone slices, are shown as 3D reconstructions from Z‐stacks. Bone is shown in blue, osteoclasts in green. Scale bars = 10 μmol/L

In the next series of experiments, human CD14^+^ PBMCs were used to investigate the effect of aminothiazoles on osteoclast formation. PBMCs were stimulated with M‐CSF (30 ng/mL) and RANKL (2 ng/mL) and with or without inhibitors TH‐848 (0.2 μmol/L) and TH‐644 (2 and 15 μmol/L) for 6‐12 days. After 6 days, RANKL stimulation induced extensive formation of TRAP‐positive multinucleated cells, which was significantly reduced by the treatment with aminothiazole TH‐848 (Figure 5A,B). The inhibitor TH‐644, at 2 μmol/L, did not affect the formation of TRAP‐positive multinucleated cells (Figure 5A,B).

To confirm that the TRAP‐positive cells were osteoclasts, bone resorption experiments were performed on bone slices for 12 days, where cells were stimulated with M‐CSF and RANKL in the presence or absence of inhibitors. Using a confocal microscope to visualise the resorption pits, a difference in bone resorption was observed in the 3D images, reconstructed from Z‐stacks, where the cells treated with aminothiazole TH‐848 formed shallower pits, as compared to the control cells treated with RANKL only (Figure 5C and Videos S1 and S2). Similar to the previous results, aminothiazole TH‐644 (2 μmol/L) did not inhibit bone resorption pits formation (Figure 5C and Video S3). As our results did not show any effect of 2 μmol/L TH‐644 on formation of osteoclasts or resorption pits, we tested higher concentration, 15 μmol/L, of this inhibitor, as this concentration was also used for RAW 264.7 cells. Similar to TH‐848, TH‐644 also inhibited formation of both osteoclasts (Figure 5D) and bone resorption pits (Figure 5E).

**Figure 6:**
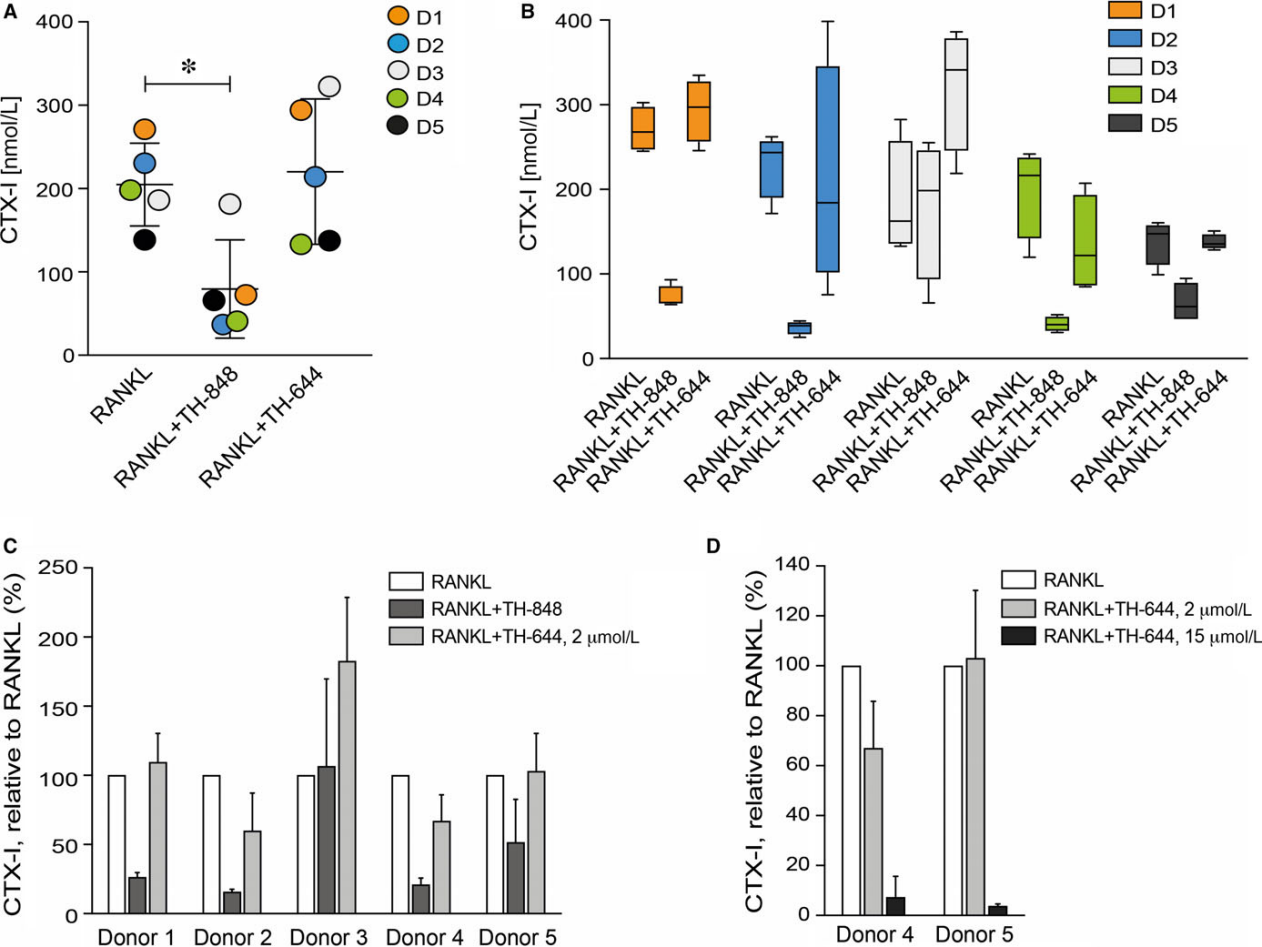
RANKL‐induced bone resorption in peripheral blood mononuclear cells (PBMCs). The CD14^+^ PBMCs were differentiated on bone slices with M‐CSF (30 ng/mL) and RANKL (2 ng/mL) alone or in the presence of aminothiazoles (A‐C) TH‐848 (0.2 μmol/L) or TH‐644 (2 μmol/L) and (D) TH‐644 (2 and 15 μmol/L) and analysed on day 9 by the CTX‐I ELISA. (A) The scatter plot shows the mean CTX‐I value of quadruplicates, where the dots represent the donors (D1‐D5) with mean±SD, *P ≤* *0.05. (B) The concentration of CTX‐I in PBMCs from the five donors, in response to RANKL with or without TH‐848 or TH‐644 treatment and (C and D) CTX‐I levels normalised to RANKL as control (100%)

To quantify bone resorption by osteoclasts, the M‐CSF‐ and RANKL‐stimulated cells were differentiated on bone slices with, or without inhibitors for 9‐12 days. Measurements of the levels of CTX‐I, a bone resorption marker, revealed that aminothiazole TH‐848 decreased CTX‐I concentration as compared to RANKL (205 and 79 nmol/L, respectively) on day 9 (Figure 6A). Comparing PBMCs from different donors, four of the five donors were responsive, to variable extent, to the inhibitor TH‐848 (Figure 6B) showing inhibition of bone resorption between 50%‐80% (Figure 6C). Treatment with aminothiazole TH‐644, did not affect CTX‐I levels at 2 μmol/L (Figure 6C), in contrast to 15 μmol/L, which almost abolished bone resorption (Figure 6D). The measurements of CTX‐I were also performed on day 12, showing similar results as above (data not shown).

## 4 DISCUSSION

Periodontitis is a chronic inflammatory disease with a multifactorial pathogenesis where bacterial load, host immune response, systemic inflammatory diseases and environmental factors, collectively contributing to the destruction of tissue and alveolar bone.^28^ In an attempt to investigate the effect of mPGES‐1 inhibitors on osteoclast formation and bone resorption, we used a co‐culture model of fibroblast‐like PDL cells and osteoclast progenitor cells RAW 264.7 as well as PBMCs stimulated by LPS or RANKL.

The results from the current study showed that LPS stimulate RAW 264.7 cells to differentiate into osteoclast‐like cells both in cell‐cell co‐cultures, co‐cultures separated by semipermeable membranes as well as in cultures of RAW 264.7 cells alone. This is in line with earlier studies showing increased formation of osteoclasts by PGE_2_ and LPS in RANKL‐treated RAW 264.7 cells.^29^ In addition, the aminothiazoles TH‐848 and TH‐644 known to inhibit PGE_2_ by targeting the synthase mPGES‐1,^22^ inhibited the LPS‐stimulated osteoclast‐like cell formation, in agreement with our recently published results.^23^ In cell‐cell co‐cultures between PDL cells and RAW 264.7 cells, a promoted formation of osteoclast‐like cells occurred compared to co‐cultures separated by semipermeable membranes. The increased numbers of osteoclast‐like cells in cell‐cell co‐cultures, compared to co‐cultures separated by a semipermeable membrane, may be explained by the fact that RANKL exists in two forms, a membrane‐bound and soluble form. Cleavage of the membrane‐bound form requires the presence of proteases, for example, matrix metalloproteinases (MMPs)^30^ and this form of RANKL is also reported to have a stronger effect on osteoclast formation than soluble RANKL.^31^ Therefore, in cell‐cell co‐cultures both forms of RANKL produced by PDL cells may be able to bind RANK on RAW 264.7 cells resulting in an increased number of osteoclast‐like cells compared to separated co‐cultures, where only the soluble form of RANKL may affect RAW 264.7 cells. In addition, in line with our results, it has been reported that direct cell‐cell contact between PDL and osteoclast progenitor cells generates a huge stimulatory effect on the expression of various genes related to osteoclast formation such as RANKL, TNF‐α and IL‐1β.^24^

Interestingly, osteoclast‐like cell differentiation was greater in LPS‐stimulated cultures of RAW 264.7 cells alone compared to co‐cultures with PDL cells. The RAW 264.7 cells are known to form multinucleated TRAP‐positive cells in response to inflammatory stimuli^32^ and this process is sensitive to RAW 264.7 cell density, which may explain the lower number of TRAP‐positive multinucleated cells formed in cell‐cell and separated co‐cultures, as compared to RAW 264.7 cultures alone. Another explanation for this might be the fact that PDL cells produce both RANKL and its decoy receptor OPG, which has been reported to be produced in considerably higher levels than RANKL in PDL cells.^3^ Thus, OPG may partly inhibit RANKL, leading to reduced formation of osteoclast‐like cells in the co‐cultures compared to RAW 264.7 cells alone. This suggestion is supported by Bloemen et al^24^ and is in agreement with our results demonstrating an overall production of OPG in the culture medium mainly from PDL cells as well as an increased mRNA expression of *OPG* by LPS in PDL cells, especially because there was no difference in OPG production between cell‐cell or separated co‐cultures. However, despite high levels of OPG, PDL cells can induce differentiation of osteoclast‐like cells, due to two‐way signalling between RAW 264.7 cells and a tight contact between cells in cell‐cell cultures, creating a favourable environment for RANKL‐RANK binding, preventing OPG to bind to RANKL^3^ and thereby leading to the inhibition of osteoclastogenesis and bone resorption.

The precise role of PDL cells in inflammatory bone loss is not fully clarified. These cells play an integral role in the production of the extracellular matrix of the PDL^33^ but apart from that, these fibroblast‐like cells have been shown to influence the migratory capacity, phagocytic activity and phenotypic maturation of the dendritic cells and macrophages.^34^ PDL cells have also been shown to up‐regulate RANKL when stimulated with PGE_2_, indicating that they are not only structural cells but also serve a regulatory role in inflammatory bone loss.^35^ In the current study, we investigated the production of PGE_2_ in response to LPS‐treatment alone or in combination with the aminothiazoles in co‐cultures as well as in cultures of PDL and RAW 264.7 cells alone to elucidate the role of PDL cells in inflammation‐induced osteoclastogenesis. Our results demonstrated that PGE_2_ levels increased in response to LPS treatment and decreased by the aminothiazoles in co‐cultures of PDL and RAW 264.7 cells as well as in these cells alone. These results correlate well with previously reported results by our group demonstrating that the aminothiazoles inhibits cytokine‐induced PGE_2_ production in gingival fibroblasts as well as in RAW 264.7 cells.^22^, ^23^ The overall PGE_2_ production in response to LPS was lower in PDL cells compared co‐cultures or RAW 264.7 cells alone, suggesting that PDL cells have a minor role contributing to the inflammation‐induced PGE_2_ production in this co‐culture model, mimicking the complex interaction between cells during inflammatory bone loss. Similar to PGE_2_, the production of the inflammatory cytokine IL‐6 was increased by LPS, although the levels of IL‐6 were not affected by aminothiazoles, highlighting the latter as specific PGE_2_ inhibitors. When comparing the overall levels of IL‐6, the greatest production was observed in cell‐cell co‐cultures followed by separated co‐cultures and the lowest concentrations were observed in PDL cells alone suggesting that RAW 264.7 cells are the main contributors to IL‐6 production in this model. Increased levels of IL‐6 in response to LPS have been reported in both PDL cells and RAW 264.7 cells,^36^, ^37^, ^38^ but to the best of our knowledge not in co‐cultures of PDL and RAW 264.7 cells, although the clinical significance of this observation remains to be clarified.

In the current study, we also investigated the effects of aminothiazoles on osteoclastogenesis and bone resorption using human PBMCs, stimulated with RANKL. Our results demonstrated that aminothiazoles (TH‐848 and TH‐644) inhibited the RANKL‐induced osteoclast differentiation. In addition, both aminothiazoles were able to prevent resorption pits formation, when RANKL‐treated PBMCs were cultured on the bone slices. Moreover, we performed the quantification of the inhibitory effect of aminothiazoles on the RANKL‐induced bone loss by the CTX‐I assay, revealing that both TH‐848 and TH‐644 reduced the levels of bone resorption marker. The inhibition of bone resorption by aminothiazoles, at the concentrations used in the current study, was higher than 50%. These results are in line with our previous findings demonstrating that the aminothiazoles decreased alveolar bone destruction, by almost 50%, in experimental periodontitis in rat model.^22^ In the current study, different concentrations of aminothiazole TH‐644 were used for different cells. The low dose (2 μmol/L) of aminothiazole was sufficient, when PDL cells were cultured alone, whereas RAW 264.7 cells required a higher dose (15 μmol/L) of TH‐644 to inhibit PGE_2_ production and osteoclast formation. This could be explained by the fact that the aminothiazoles were identified based on their ability to bind to the active site of the human mPGES‐1 enzyme,^22^ and that RAW 264.7 cells are of murine origin. TH‐644 may have a lower binding to the murine orthologue of mPGES‐1 and higher concentrations were therefore required to obtain an inhibitory effect, as it has been reported that there are structural differences between human and rodent mPGES‐1.^39^, ^40^ However, the higher dose of TH‐644 was also required for PBMCs cultures, as no effect could be observed by 2 μmol/L. In contrast to TH‐644, TH‐848 inhibitor was more potent, as it inhibited PGE_2_ production and osteoclast formation at much lower concentrations in RAW 264.7 cells, as well as osteoclast formation and bone resorption in human PBMCs, indicating that the potency of this inhibitor seems to be independent of the origin of cells. This notion is also supported by our previous results demonstrating reduced alveolar bone loss in experimental‐induced periodontitis in rats as well as inhibition of PGE_2_ in human gingival fibroblasts, rat gingival fibroblasts and RAW 264.7 cells.^22^, ^23^

The RAW 264.7 cells form osteoclast‐like cells in response to both RANKL and LPS,^23^, ^32^ which is inhibited by aminothiazoles. Furthermore, in human PBMCs, these aminothiazoles, targeting mPGES‐1, reduced the LPS‐induced PGE_2_ production^23^ as well as inhibited the RANKL‐stimulated osteoclastogenesis and bone resorption in the current study. Another study by Tominari et al^41^ showed that osteoclast differentiation from RANKL‐stimulated mouse bone marrow macrophages is suppressed by compounds inhibiting PGE_2_ synthesis by targeting both COX‐2 and mPGES‐1. Both RANKL‐RANK^42^ and LPS‐TLR4^43^ signalling pathways are mediated via transcription factor NF‐κB. In response to stimuli, the IKKα/IKKβ/NEMO (IKKs) complex is activated, leading to dissociation of IκBα/NF‐κB complex, and subsequently to nuclear translocation of NF‐κB.^44^ PGE_2_‐ and RANKL‐mediated signalling converge at the Akt‐, cAMP‐Ca^2+^‐calcineurin‐NFATc1‐45 and Wnt/β‐catenin pathways,^42^ and therefore both may be sensitive to aminothazoles. This suggestion is further supported by the findings that the expression of NFATc1, its binding and transcriptional activity of NFAT is reduced by antagonists of PGE_2_ receptors^46^ that are known to play important role in regulating bone formation and resorption.^45^, ^47^ Furthermore, RANK‐RANKL signalling pathway acting through NF‐kB, MAPKs and AKT, induces the expression of different osteoclast‐specific genes, including TRAP, MMP‐9 and cathepsin K (CTSK), leading to osteoclast differentiation and bone resorption.^48^, ^49^ It is noteworthy to mention that the levels of CTSK, the major protease in bone resorption, are reduced by aminothiazoles in LPS as well as RANKL‐stimulated RAW 264.7 cells.^23^

Periodontitis is a common chronic inflammatory disease resulting in the loss of alveolar bone supporting the teeth leading to tooth loss. Inhibitors of mPGES‐1, including aminothiazoles, are relatively new compounds included to the group of substances with anti‐inflammatory properties. To our knowledge, up to date there are no mPGES‐1 inhibitors approved for clinical use. Our group is the first to report and elucidate the impact of aminothiazoles as mPGES‐1 inhibitors in a model of periodontitis in vitro.^22^ The findings that aminothiazoles inhibit osteoclast differentiation and bone resorption in cell types tested, suggest these compounds as potential inhibitors of bone loss.

## 5 CONCLUSIONS

In conclusion, the results from this study that aminothiazoles successfully inhibited in vitro PGE_2_‐production, osteoclastogenesis and bone resorption, suggesting these inhibitors to be a novel aid in future treatment of inflammatory bone loss such as periodontitis.

## CONFLICT OF INTEREST

The authors declare that there are no conflicts of interest.
