# Supplementary material for: Aminothiazoles inhibit osteoclastogenesis and PGE 2 production in LPS‐stimulated co‐cultures of periodontal ligament and RAW 264.7 cells, and RANKL‐mediated osteoclastogenesis and bone resorption in PBMCs
Source: J Cell Mol Med. 2018 Dec 1;23(2):1152–63. doi: 10.1111/jcmm.14015 (PMC6349150; doi:10.1111/jcmm.14015)
Supplement: Supplementary file 1 [file JCMM-23-1152-s001.pdf]

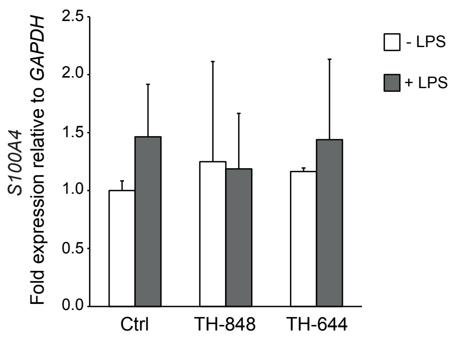

Figure S1. The periodontal ligament (PDL) cell marker *S100A4* mRNA expression in PDL cells. Cultures were treated for 24 hr with or without lipopolysaccharide (LPS; 1 μg/ml) in the absence or presence of the aminothiazoles TH-848 (0.2 μM) or TH-644 (2 μM). The results show relative mRNA expression normalized to *GAPDH* ± SD and represents the mean of three independent experiments.
