# Supplementary material for: Aminothiazoles inhibit osteoclastogenesis and PGE 2 production in LPS‐stimulated co‐cultures of periodontal ligament and RAW 264.7 cells, and RANKL‐mediated osteoclastogenesis and bone resorption in PBMCs
Source: J Cell Mol Med. 2018 Dec 1;23(2):1152–63. doi: 10.1111/jcmm.14015 (PMC6349150; doi:10.1111/jcmm.14015)
Supplement: Supplementary file 6 [file JCMM-23-1152-s006.pdf]

TRAP staining

For TRAP staining of the co-cultures, cells were washed twice with 0.15M NaCl solution, thereafter fixed in formaldehyde 2% for at least 30 min and then washed again twice in physiological NaCl-solution. The cells were stained for TRAP using the commercial Acid Phosphatase, Leukocyte (TRAP) kit (Sigma-Aldrich, MO, USA) according to the instructions provided by the manufacturer. TRAP-positive multinucleated cells containing ≥3 nuclei were counted as osteoclast-like cells. The counting of a predefined area was performed under a light microscope, using 4x objective by two calibrated independent observers.

For TRAP staining of the osteoclast cultures, cells were washed with PBS, fixed with mixture of acetone and 3% formaldehyde in citrate buffer at room temperature for 30 sec and washed thereafter with H_2O. The cells were then stained for phosphatase activity using TRAP kit, according to manufacturer's instructions. Images were taken using 4x objective with Nikon Eclipse TE300 microscope (Nikon) and RGB colour CCD camera. Quantification of the TRAP-positive osteoclasts was performed using ImageJ macro software (NIH) from 4x images by counting cells larger than mononuclear cells (area > 215 μm^2) with high TRAP-staining intensity.

The code for the quantification of the multinuclear TRAP-positive cells (osteoclasts):

```
Stack.setDisplayMode("grayscale");
run("Z Project...", "start=2 projection=[Sum Slices]");
rename("SUM_ActiveR+G.jp2");
selectWindow("SUM_ActiveR+G.jp2");
run("Median...", "radius=1");
run("8-bit");
setThreshold(0, 45);
run("Analyze Particles...", "size=40-Infinity show=Outlines display include");
%The 40 pixel area corresponds to area of 215 μm2 and average size of a mononuclear cell is below 200 μm2
String.copyResults();
```

Supplementary Video 1. Z-stack of bone resorption pits formed by osteoclasts, differentiated for 12 days on the bone slice with M-CSF (30 ng/ml) and RANKL (2 ng/ml). Result is representative from 5 different PBMCs. Bone is shown in white, osteoclasts - in magenta. Scale bar = 20 µm.

Supplementary Video 2. Z-stack of bone resorption pits formed by osteoclasts, differentiated for 12 days on the bone slice with M-CSF (30 ng/ml) and RANKL (2 ng/ml) in the presence of aminothiazole TH-848 (0.2 µM). Result is representative from 5 different PBMCs. Bone is shown in white, osteoclasts - in magenta. Scale bar = 20 µm.

Supplementary Video 3. Z-stack of bone resorption pits formed by osteoclasts, differentiated for 12 days on the bone slice with M-CSF (30 ng/ml) and RANKL (2 ng/ml) in the presence of aminothiazole TH-644 (2 µM). Result is representative from 5 different PBMCs. Bone is shown in white, osteoclasts - in magenta. Scale bar = 20 µm.

Supplementary Video 4. Z-stack of bone resorption pits formed by osteoclasts, differentiated for 12 days on the bone slice with M-CSF (30 ng/ml) and RANKL (2 ng/ml) in the presence of aminothiazole TH-644 (15 µM). Result is representative from 2 different PBMCs. Bone is shown in white, osteoclasts - in magenta. Scale bar = 20 µm.
